# Assessing the perceptions of a biostatistics and epidemiology module: Views of Year 2 medical students from a Malaysian university. A cross-sectional survey

**DOI:** 10.1186/1472-6920-10-34

**Published:** 2010-05-13

**Authors:** Aqil M Daher, Farzana Amin

**Affiliations:** 1 Department of Population Health and Preventive Medicine, Faculty of Medicine, Universiti Teknology MARA(UiTM), Shah Alam, Selangor, Malaysia

## Abstract

**Background:**

In the era of evidence based medicine, biostatistics and epidemiology are considered as the main elements aiding the health professional to design a research study, understand the literature, and make decisions about patient care. The aim of the study is to explore students' perception about this subject because it plays an important role in determining educational outcome.

**Methods:**

Data were collected from a self-administered questionnaire distributed among 164 Year 2 medical students. The 5-point Likert scale anchored by Strongly disagree = 1 and Strongly agree = 5 included 36 questions in four domains designed to assess the perception of a biostatistics and epidemiology module amongst students.

**Results:**

138 students with ages ranging from 20 to 24 years (Mean = 20.7; SD = 0.62) returned their responses to the questionnaire. This was a response rate of 84.14%. Of the 138 students, 80.7% realized the relevance of the subject to real health issues at the end of the module, while 89.8% believed the module focused on interpretation more than calculation.

More than three quarters (78.1%) agreed that lack of practicing exercises was the cause for declining interest in the subject, while only 26.1% believed that lectures were not interesting. Another three quarters (75.4%) believed that there were too many lectures for one day of teaching activities, while 84.6% recommended practical sessions for designing research and data collection.

**Conclusions:**

This study found that students perceived the relevance of biostatistics and epidemiology to real health issues. The major cause of poor interest in the subject was attributed to the short duration of the course, lack of practicing exercises, and the need for practical data collection sessions. Emphasis should be given to early introduction of projects for data collection and analysis.

## Background

Ongoing advances in knowledge and technology in healthcare has offered new and better ways to solve the key health problems. On the other hand, increasing volume and diversity of information, controversies, and complexities, particularly with the increasing cost of medical care, has required greater knowledge in order to make decisions about the care of individual patients or the delivery of health services.

Evidence based medicine has reduced the emphasis on unsystematic clinical experience and pathophysiological rationale, and instead focused attention on critical appraisal of clinical research, which is considered the source of the evidence [1].

For health professionals to be able to fully understand the results of research, and to make decisions after critically reviewing the evidence, they need to be equipped with knowledge of biostatistics and epidemiology. Moreover, reading literature and identifying serious flaws in design, analyses and interpretation begins early in the training of health professionals and continues throughout their careers [2,3].

A clear statement was made during the Edinburgh Declaration of the World Medical Association in 1988; the purpose of the medical education is to produce professionals who are able to understand the needs of their communities and respond accordingly [4]. This requires physicians who are familiar with the community and its health problems and know what is required for their prevention and solutions [5].

To achieve this, medical students must gain professional skills and theoretical knowledge in how to obtain the reference ranges of "normal values" for various biochemical and physiological measures. These measures are used to make the diagnosis and identify diseased from healthy individuals. Additionally, determining test validity, designing research, and drawing inference requires knowledge of sampling, and calculation of prevalence, rate and proportion, and risk. Without this a doctor may draw disastrous conclusions from his/her clinical experience because he/she has no concept of appropriate scientific method.

The role of biostatistics and epidemiology is well recognized in the curricula of medical schools in developed and developing countries but with great variability from school to school in terms of time allocated, scope, and depth of topics covered. For instance in the majority of medical schools in the USA, biostatistics, which also includes related subjects such as epidemiology, preventive medicine demography, and medical informatics, is taught during the first or second year over more than one term as a component of the public health training [6]. In Ziauddin university in Pakistan, epidemiology, biostatistics, and survey methodology courses are taught in the first two years of medical school [7]. In South Africa biostatistics and research methodology are taught in year 1 and 2 with further reinforcement practiced in year 3 and 4 in some universities [8], and in 44 medical schools in Turkey, biostatistics is usually taught in the 1st year [9].

Outcome based medical education could be viewed as the modern model that focuses on measuring student performance rather than on the resources available to the student [10,11]. Several studies in the field have emphasized the decisive role that students' perceptions and attitudes about a subject play in the achievement of educational outcomes [12-14]. Perception is defined as the process of attaining awareness or understanding of sensory information, which an individual exhibits towards certain processes, situations, objects or persons [15].

Biostatistics and epidemiology are taught together in year 2 at Universiti Teknology MARA (UiTM) as a short course of 4 weeks. The short course is offered as a communicable disease module under the population health and preventive medicine discipline. The objectives of this course are (1) to enable medical students to understand the language and principles of biostatistics and epidemiology, (2) to highlight the nature and the distribution of disease(s), and (3) to teach students to design their own research projects, as well as to be able to critically read and understand scientific papers in medical journals. Thus it covers basic theoretical concepts about epidemiology and biostatistics along with hands-on sessions of computer application software. The software includes Statistical Package for Social Sciences (SPSS), Power and Sample Size Calculation (PS), and Epi Info. In this module emphasis was given to the understanding of concepts rather than carrying out routine statistical computations.

The module is composed of formal lectures, computer lab sessions for hands-on exercise, directed self learning sessions, problem based learning sessions, and two movies about outbreak investigation and control. The sequence of the topics was carefully observed so that epidemiology and biostatistics were taught in a parallel manner to amalgamate the knowledge of the two topics. Each lecture of biostatistics was followed by a hands-on session when applicable. The hands-on sessions are to train students to do basic statistical analyses using computer software. At the end of each week of the module the students undertake a directed self-learning session. The session is a scenario of a common health problem that includes topics taught in that specific week, and based on that scenario the students have to answer a number of questions that integrate knowledge of epidemiology and biostatistics. Thus, in the same question there may be epidemiological and statistical concepts. Problem based learning sessions are meant to integrate other basic science knowledge in the context of epidemiology and biostatics. "See Additional file 1"

The main objective of the current study was to assess the perceptions of Year 2 medical students towards the Biostatistics and Epidemiology module. Statistics in most literature is reported to be the cause of negative perception [16-19]. In spite of this, we could not separate biostatistics and epidemiology in the questionnaire because the syllabus is designed in such a way that the two are complemented. We propose that an understanding of student's perceptions provides important elements in the development of enhanced educational strategies leading to better learning outcomes.

## Methods

### Participant

The study targeted Year 2 medical students at UiTM in the 2008-2009 academic session. A total of 164 students, 48 (29.2%) males and 116 (70.8%) females were targeted in the study employing universal sampling. The students came from two different educational backgrounds. The first group of students came from a matriculation institute while the second group was from a science background. Students in both educational backgrounds had been taught biology, chemistry, physics and mathematics.

### **Instrument and procedure**

To develop a questionnaire for this study, the researchers initially reviewed literature pertaining to attitudes and perceptions of medical students in general, as well as epidemiology and biostatistics in particular. This process was followed by a focus group discussion. The participants of this discussion were professors of the subject, a medical education expert, a psychologist and students. The focus group was constituted in this manner in order to ensure that relevant stakeholders had a say in the contents of the questionnaire. This was also important in ensuring the face validity of the questionnaire. To measure the perceptions of medical students a Likert scale anchored by 1 = Strongly disagree and 5 = Strongly agree was developed. The researcher felt the use of such a scale would be relatively easy, and the interpretation of the results straightforward [20].

As a pilot, the first draft was given to a sample of 20 students to ensure face validity, to assess comprehension of the questionnaire, and to take into consideration any comments provided by the students. The resulting questionnaire had 36 items. Participants were also requested to provide information relating to their age, sex, background and possession of laptop and computer software.

The 36 questions assessing perceptions toward the course fell into four domains, namely (A) Course Value, (B) Difficulties, (C) Behavior, and (D) Expectation. "See Additional file 2".

The Course Value domain was about perceptions of the usefulness, relevance and worth of the subject in professional life. A typical item statement in this domain was "I realized the relevance of epidemiology and statistics to the real health issues". The Difficulties domain was about difficulties faced by students and factors that might influence interest in the subject. A typical item statement was "Lack of practicing exercise for these topics and lectures were difficult to understand". The Behavior domain was about how the students perceived lecturer behavior towards them. The typical item statement in this domain was "Work and efforts were acknowledged". The Expectation domain was about possible actions that may influence the outcome of the course. The typical item statement was "Need more practical workshops for planning and data collection to have real experience in dealing with data". The internal consistency of the scale items is shown in Table 1.

**Table 1:** Cronbach's alpha for internal consistency

Domain	Number of item	Cronbach's alpha
A Course Value	9	0.824

B Difficulties	11	0.661

C Behavioral	5	0.785

D Expectation	11	0.610

Total	36	0.708

The aim of the study was explained to the students, and they were informed that participation was voluntary and the results would remain anonymous. The questionnaire was administered in April 2009 after completion of the communicable disease module during the first session of Problem Based Learning of the subsequent module.

### **Statistical analyses**

Data were entered and analyzed using SPSS version 16. Categorical variables were described by frequencies and percentage, and numerical variables with mean and standard deviation.

Independent samples t-test was used to compare the mean domain score for each independent variable. Significance level was set at 0.05.

### Scoring

To categorize the score of each domain into negative-fair perceptions and positive perception, a criterion was established. We considered the value of more than 70% of the total domain score to represent positive perceptions, calculated as follows:

Number of domain variables * 5* 0.7

## Results

Overall the questionnaire has a satisfactory level of consistency, reflected by the given reliability coefficient [21]. Items of the Course Value domain showed the highest consistency, while the Expectation domain showed the lowest value. (Table 1)

The characteristics of the study sample are shown in Table 2. A total of 138 out of 164 students responded to the questionnaire yielding a response rate of 84.14%. The mean age was 20.7 year with a standard deviation (SD) of 0.6 years. With respect to sex, there were 41 (29.7%) males and 97 (70.3%) females. There was no gender difference in the response rate. About four fifths (79.9%) of students had a personal computer, while only 19.6% had licensed SPSS computer software. The possession of free PS and Epi Info software was reported by 18.1% and 24.6%, respectively.

**Table 2:** characteristics of study sample

Variable		Frequency	Percent
**Sex**			

	Male	41	29.7

	Female	97	70.3

**Pre-college educational institute**			

	Matriculation institute	89	66.9

	Pre Science Degree	44	33.1

**Has personal computer**			

	No	28	20.3

	Yes	110	79.7

**Has SPSS software**			

	No	111	80.4

	Yes	27	19.6

**Has PS (Power and Sample) software**			

	No	113	81.9

	Yes	25	18.1

**Has Epi Info software**			

	No	104	75.4

	Yes	34	24.6

**Age **Mean(SD)		20.73(0.62)	

Responses to each survey question are presented in Table 3. Regarding the Course Value domain, it may be observed that 80.7% of respondents realized the relevance of the subject to the real health issues at the end of module, while only 32.6% felt that they were confident to do basic statistical and epidemiological analysis and 37.0% felt they gained skill in designing research. With respect to the Difficulties domain, 78.1% of the respondents indicated that the lack of practicing exercises was the cause for declining interest in the subject, while only 26.1% believed that lectures were not interesting. About 58.7% indicated more interest in clinical studies than these topics and 31.6% were not interested in the subject.

**Table 3:** response to each question ...

Question	Strongly disagree-Disagree	Neutral	Agree-Strongly agree
**Domain A **: **Course Value **n(%)			

The course focuses on the concept of interpretation more than calculations.	7(5.1)	7(5.1)	123(89.8)

I realized the relevance of Epidemiology & Statistics to the real health issues.	10(7.4)	16(11.9)	109(80.7)

Sequencing of topics was logical.	15(10.9)	18(13.1)	104(75.9)

The gained knowledge and experience is useful to my career as a doctor.	7(5.1)	32(23.4)	98(71.5)

I understood the main concepts of Epidemiology & Statistics.	27(20.0)	34(25.2)	74(54.8)

I gained skills to read scientific papers.	22(16.3)	41(30.4)	72(53.3)

My skills improved in solving problems.	32(23.7)	51(37.8)	52(38.5)

I gained skills to design research	32(23.7)	53(39.3)	50(37.0)

I gained confidence in my ability to do basic statistical & epidemiological analysis.	41(30.4)	50(37.0)	44(32.6)

**Domain B: Difficulties **n(%)			

Lack of practicing exercise for these topics	15(10.9)	15(10.9)	107(78.1)

Too many lectures for one day	9(6.5)	25(18.1)	104(75.4)

Subjects need creative thinking.	25(18.2)	22(16.1)	90(65.7)

Lectures were difficult to understand	24(17.4)	29(21.0)	85(61.6)

I like clinical studies more than epidemiology and biostatistics	16(11.6)	39(28.3)	81(58.7)

Lectures were lengthy	24(17.4)	37(26.8)	77(55.8)

There were no specific references	43(31.4)	23(16.8)	71(51.8)

I have to deal with numbers	64(46.7)	25(18.2)	48(35.0)

Simply am not interested in the subject	58(42.6)	35(25.7)	43(31.6)

I could not see the relation between statistics and medicine at this level.	65(47.1)	30(21.7)	43(31.2)

Lectures were not interesting	66(47.8)	36(26.1)	36(26.1)

**Domain C: Behavioral **n(%)			

Lecturer is the facilitator of instruction & guiding students	0	3(2.2)	134(97.8)

Lecturer is the source of knowledge	1(0.7)	3(2.2)	134(97.1)

I was treated with respect	1(0.7)	14(10.3)	121(89)

Work and efforts were acknowledged	8(5.8)	16(11.7)	113(82.5)

It is the responsibility of the student to initiate debate/question during lectures.	3(2.2)	30(21.7)	105(76.1)

**Domain D: Expectations **n(%)			

Need More practical, workshop for planning and data collection to have real experience in dealing with data	5(3.7)	16(11.8)	115(84.6)

Provide specific text books for biostatistics and epidemiology	6(4.4)	16(11.8)	114(83.8)

Carry out shorts exam (quiz) before the progress test to evaluate the understanding of the student	6(4.4)	21(15.4)	109(80.1)

The lecture should be followed by Small Group Session	12(9)	23(17.3)	98(73.7)

Give more time for the whole course	22(16.4)	18(13.4)	94(70.1)

Emphasize on using biostatistics in elective courses	12(8.8)	36(26.5)	88(64.7)

Attendance to be strictly taken during the computer lab session	25(18.4)	26(19.1)	85(62.5)

Make the module pure for biostatistics and epidemiology, so the attention will not be withdrawn to other subjects	28(20.6)	28(20.6)	80(58.8)

Introduce this course earlier in year two	24(17.6)	33(24.3)	79(58.1)

I have to study at home before class meetings	14(10.4)	49(36.3)	72(53.3)

Disconnect the internet during the lab session to avoid distraction	41(30.1)	30(22.1)	65(47.8)

Concerning the Behavior domain, 97.1% believed that the lecturer was the source of knowledge, and 76.1% deem it is the student's responsibility to initiate debate during the class. In the Expectations Domain, 84.6% recommended practical sessions for designing research and data collection. Another 73.7% emphasized that the lecture should be followed by a small group session. On the other hand, only 53.3% believed that they need to prepare before the class meeting. Notably 70.1% required more time for the whole course.

Overall, it is observable from Table 4 that most of the students were categorized as reflecting positive perceptions. The exception however was with the Difficulties domain where students were categorized as indicating negative perceptions.

**Table 4:** frequency distribution of positive perception

	Positive perception	
**Domain**	**No**	**Yes**

A: Course Value	46(33.3)	92(66.7)

B:Difficulties	85(61.6)	53(38.4)

C: Behavioral	11(8.0)	127(92.0)

D: Expectation	47(34.3)	90(65.7)

In general the differences in mean score were not statistically significant in each of the 4 domains scored when grouped by gender, pre-college educational background, and possession of personal computer and software(table 5). Even when it was marginally significant the difference was too small to be of practical relevance (small effect size) [22].

**Table 5:** The mean domain score by selected independent variables.

Independent variable		N	Domain A		Domain B		Domain C		Domain D	
			
			Mean(SD)	P *	Mean(SD)	P *	Mean(SD)	P *	Mean(SD)	P *
Sex	Male	41	31.9 (5.8)		37.3(6.2)		20.9 (2.5)		40.9 (4.9)	
				0.49		0.30		0.52		0.78
	female	97	31.1 (6.3)		36.2 (5.8)		20.6 (2.5)		40.6(6.3)	

Pre-college educational institute	Matriculation institute	89	31.9 (5.5)		36.5 (5.6)		20.8 (2.4)		40.3 (5.1)	
				0.14		0.84		0.48		0.05
	Pre Science Degree	44	30.2 (7.7)		36.3(6.5)		20.5 (2.8)		38.7 (6.8)	

Has personal computer	No	28	31.4 (6.3)		36.1(5.4)		20.7(2.7)		40.9(5.1)	
				0.99		0.42		0.98		0.85
	Yes	110	31.3 (6.2)		35.9(5.9)		20.7(2.5)		40.6(6.1)	

Has SPSS software	No	111	31.1 (6.3)		36.8(5.9)		20. 8(2.5)		40.6(5.9)	
				0.27		0.25		0.52		0.63
	Yes	27	32.5 (5.5)		35. 4(5.5)		20.4(2.7)		41.1(5.9)	

Has Power and Sample software	No	113	30.7 (6.5)		36.9(6.1)		20.7 (2.5)		40.5(5.9)	
				0.19		0.0.7		0.65		0.46
	Yes	25	32.5 (2.7)		34.6(4. 8)		20.5(2. 6)		41.5(5.4)	

Has Epi Info software	No	104	31.8 (6.5)		35.2(5.9)		20.5(2.5)		40. 4(6.1)	
				0.06		0.17		0.45		0.28
	Yes	34	33.2(3.6)		34.4(5.5)		20.9(2. 5)		41.6(5.3)	

*: P value for independent samples t test is not significant at 0.05

Domain A = Course Value, Domain B = Difficulties, Domain C = Behavioral and Domain D = Expectation

## Discussion

Here we report the results of the first study in a Malaysian medical institute about students' perception toward biostatistics and epidemiology. These results contradict the belief that the significance of biostatistics in Medicine and Health Care is often only appreciated by qualified medical practitioners [18]. To the contrary, the results of this study suggested that more than half of the students had a positive perception about the course value (i.e. this course is beneficial for their career and they could feel the link of biostatistics and epidemiology to real health issues). This may be due to the content of this course which is highly focused and was taught purely by specialized medical doctors in epidemiology and biostatistics who could relate the two aspects of health. A similar finding was reported from a Pakistani medical institute, where the majority of students surveyed showed a positive response regarding the relevance of biostatistics and epidemiology to medical curriculum [7]. A researcher from Croatia also reported positive attitudes about science and scientific research in medicine among undergraduates [23].

It was previously reported that biostatistics is one of the subjects in the medical curriculum that is potentially disliked by the majority of undergraduate students, most likely because it encompasses mathematics and calculation which can cause confusion [17,18,24]. The result of our study showed students perceived "mathematical calculation" of secondary importance since the focus of the course was on interpretation of the results and medical literature.

Among the perceived difficulties by students, which may affect the outcome of the course, is the lack of practicing exercise for the topic. This was attributed to the short duration of the course which included many activities per day. A finding that was consistent in a high proportion of students regarding the perceived difficulties and possible solutions was the recommendation to give more time for the course, designate a small group session following each lecture, and to introduce practical sessions of data collection and analysis. An American study had shown that students' early participation in research activity improve their knowledge and attitude towards research [25].

The finding that students found lectures difficult to understand may be attributed to the teaching activities used, prior exposure of the student to biostatistics and epidemiology, and the experience of the lecturer. Instructors have diverse background and varying talents in terms of teaching skills, degree of emphasis and approach of teaching. Each has his or her own style, strength, weakness, and vision of how the instruction should be. Shift of interest to clinical studies may be explained by the fact medical students do not envision a career as investigator; they tend to concentrate on more traditional medicine subjects. This shift is also attributed to the fact that statistics is a different subject from those on which the students spend most of their time [26].

Although the learning process is student centered, the majority of the study sample perceived that the lecturer is the main source of knowledge, perhaps due to the students' perceived lack of resources, difficulty to understand the textbooks on such topics, and the short time available for study. These factors could result in the belief that it is better to depend on what the lecturers provide. On the other hand students perceived a good attitude from the lecturers toward them, reflecting the awareness of the lecturers on the role of social support in motivating students and improving educational outcome.

We expected that possession of a personal computer and licensed software could influence the perception because they provide additional opportunity for learning outside of class time, but results did not show a significant effect.

We recognize several limitations of this study. First, although efforts were made by the authors to explain the explorative nature of the study and that it bears no relation to the students' academic performance, there are still some concerns about the possible effect of student-lecturer relationships on reported students' perception. Such a bias would fall in the direction of overestimating positive perceptions. Second, there was no comparison to the perception of the course at another institute in a different setting. Finally, there were no open comments requested. However, such open comments require different methods of qualitative analyses.

## Conclusions

Epidemiology and biostatics are important subjects in the medical curriculum and are closely related to health care. Short course durations may prohibit a comprehensive explanation of the topic and may limit the students' participation and/or understanding.

Lack of practical exercises and the need for data collection sessions are the major challenges faced by the students. Actions directed toward these challenges may involve spreading the module over a longer time period with increased time allotted for the module. Introducing data collection sessions and reading excerpts from published medical articles will provide practical experience and emphasize the role of biostatistics and epidemiology in health care.

Other learning methods may involve the use of video films and other visual aids to clarify and reinforce a variety of statistical concepts, motivate the study of a new topic, and to make statistics an interesting and exciting subject.

## Competing interests

The authors declare that they have no competing interests.

## Authors' contributions

The two authors contributed to development of the research idea and designing the questionnaire, carrying out data collection, data analysis, drafting and finalizing the manuscript.

## Authors' information

Both authors have a Bachelor degree of Medicine and Surgery and Master of Public Health. AMD is currently lecturer of biostatistics and epidemiology and PhD Candidate and FA is lecturer of public health at department of population health and preventive medicine, Faculty of Medicine, Universiti Teknology MARA, 40450 Shah Alam, Selangor Malaysia.

## Pre-publication history

The pre-publication history for this paper can be accessed here:

http://www.biomedcentral.com/1472-6920/10/34/prepub

## Supplementary Material

Additional file 1 **Communicable disease module lesson plan**. The table provides insight about the content of the module including the topics and duration of contact hours. Click here for file

Additional file 2 **Questionnaire on Perception of Medical Student about epidemiology & biostatistics**. The file includes the questionnaire used in the study. Click here for file
