# Supplementary material for: Assessing the perceptions of a biostatistics and epidemiology module: Views of Year 2 medical students from a Malaysian university. A cross-sectional survey
Source: BMC Med Educ. 2010 May 13;10:34. doi: 10.1186/1472-6920-10-34 (PMC2885405; doi:10.1186/1472-6920-10-34)
Supplement: Additional file 1 — Communicable disease module lesson plan. The table provides insight about the content of the module including the topics and duration of contact hours. [file 1472-6920-10-34-S1.DOC]

**Communicable disease module lesson plan**

**Population Health & Preventive Medicine**

| Topic | Title | Duration-hour |
| --- | --- | --- |
| 1 | [Principles and practice of epidemiology; Dynamics of Disease Transmission.](#__RefHeading___Toc218675760) | 2 |
| 2 | [Introduction to Statistics: Statistics & Health Sciences](#__RefHeading___Toc218675787) | 1 |
| 3 | [Descriptive Statistics](#__RefHeading___Toc218675788) | 1 |
| 4 | [Measures of Central Tendency](#__RefHeading___Toc218675789) | 1 |
| 5 | [Measures of Dispersion](#__RefHeading___Toc218675791) | 1 |
| 6 | [Intro to SPSS (Hands-on/ computer lab)](#__RefHeading___Toc218675790) | 1.5 |
| 7 | [Principles of Disease Prevention](#__RefHeading___Toc218675761) | 1 |
| 8 & 9 | [Measures of disease frequency: Morbidity & Mortality; Measures of Risk & Association.](#__RefHeading___Toc218675762) | 2 |
| 10 | [Data entry in SPSS(](#__RefHeading___Toc218675792) Hands-on exercise/ computer lab) | 1.5 |
| 11 | [Surveillance of Communicable Diseases](#__RefHeading___Toc218675765) | 1 |
| 12 | [Epidemiology of Food and Waterborne Diseases](#__RefHeading___Toc218675770) | 1 |
| 13 | Directed Self Learning | 2 |
| 14 | Investigation and control of an epidemic | 1 |
| 15 | Epidemiology and control of Vector-borne Diseases | 2.5 |
| 16 | [Introduction to Probability](#__RefHeading___Toc218675793) | 1 |
| 17 | [Probability Distributions](#__RefHeading___Toc218675794) | 1 |
| 18 | Movie 1[“Outbreak”](#__RefHeading___Toc218675764) | 2 |
| 19 | Estimation | 1 |
| 20 | Hands-on exercises/ computer lab | 1 |
| 21 | Problem Based Learning | 2 |
| 9 | [Vital statistics](#__RefHeading___Toc218675768) | 1 |
| 10 | [Screening of Diseases](#__RefHeading___Toc218675769) | 1 |
| 22 | Directed Self Learning | 2 |
| 15 | [Epidemiological Studies 1](#__RefHeading___Toc218675774) | 2 |
| 12 | [Standardization of Rates](#__RefHeading___Toc218675771) | 1 |
| 14 | Movie 2 [“Black Death”](#__RefHeading___Toc218675773) | 2 |
| 11,12 & 13 | [Sampling Methods & Sample Size Calculation](#__RefHeading___Toc218675797)- | 2 |
|  | Hands-on exercises/ computer lab | 2 |
| 16 | [Epidemiological Studies 2](#__RefHeading___Toc218675775) | 2 |
| 14 | [Statistical inference/ Hypothesis testing](#__RefHeading___Toc218675799) | 1 |
| 15&16 | Statistical Tests for Quantitative Data I : t test | 1 |
|  | Hands-on exercises/ computer lab | 1.5 |
| 17 | [Epidemiology of New and Re-emerging Diseases](#__RefHeading___Toc218675776) | 2 |
| 18 | Epidemiology of Leprosy | 1 |
| 21 | Problem Based Learning | 2 |
| 19 | [Epidemiology of TB](#__RefHeading___Toc218675778) | 1 |
| 20 | [AIDS/STD Control](#__RefHeading___Toc218675779) | 1 |
|  | Directed Self Learning | 2 |
| 17 | Statistical tests for quantitative data II: ANOVA exercises | 1 |
| 18 | Hands-on exercises/ computer lab | 1.5 |
| 21 | [Epidemiology and prevention of common cancers in Malaysia](#__RefHeading___Toc218675780) | 1 |
| 19 | Statistical Tests for Qualitative Data | 1 |
| 20 | Hands-on exercises/ computer lab | 1.5 |
| 22 | [Substance abuse Tobacco control](#__RefHeading___Toc218675782) | 1 |
| 23 | [Control of Zoonotic Diseases](#__RefHeading___Toc218675783) | 1 |
| 21 | Regression and Correlation | 2 |
| 22 | Hands-on exercises/ computer lab | 2 |
| 24 | [Immunization in Malaysia](#__RefHeading___Toc218675784) | 1 |
| 25 | [Causality in Epidemiology](#__RefHeading___Toc218675777) | 1 |
| 26 | Research Data analysis using Epi Info ( Hands-on exercises/ computer lab ) | 2 |
| 23 | Stats Review ( Hands-on exercises/ computer lab) | 2 |
| **Total** |  | **73** |
