# Supplementary material for: Assessing the perceptions of a biostatistics and epidemiology module: Views of Year 2 medical students from a Malaysian university. A cross-sectional survey
Source: BMC Med Educ. 2010 May 13;10:34. doi: 10.1186/1472-6920-10-34 (PMC2885405; doi:10.1186/1472-6920-10-34)
Supplement: Additional file 2 — Questionnaire on Perception of Medical Student about epidemiology & biostatistics. The file includes the questionnaire used in the study. [file 1472-6920-10-34-S2.DOC]

**Dear student**

We would highly appreciate your kind cooperation to respond to this questionnaire that assesses your perception on the subject of epidemiology and biostatistics Studies. Your cooperation will help us to identify problems and solutions to improve the conduct of the subject. Your response will be treated with utmost confidentiality. There are no wrong or correct responses, please give your honest responses.

Return back this questionnaire to your group leader as soon as possible.

Code number:

Age: ………………………………………….….. Gender: Male Female

Pre-college educational institute: Matriculation institute Pre-science degree

1. Do you have personal computer/laptop? **No Yes**
2. Do you have SPSS software (**S**tatistical **P**ackage for **S**ocial **S**cience)? **No Yes**
3. Do you have PS (Power and **S**ample) software?  **No Yes**
4. Do you have EpiInfo software? **No Yes**

The answer for the following questions will be indicated according to Likert scale:

**Strongly disagree (SD) Disagree(D) Neutral(N) Agree(A) Strongly Agree (SA)**

Please tick with ***√***

| **A** | **I feel that** | **SD** | **D** | **N** | **A** | **SA** |
| --- | --- | --- | --- | --- | --- | --- |
| 1. | The course focuses on the concept of interpretation more than calculations. |  |  |  |  |  |
| 2. | The gained knowledge and experience is useful to my career as a doctor. |  |  |  |  |  |
| 3. | Sequencing of topics was logical. |  |  |  |  |  |
| B | **Causes of no interest in the subject** | **SD** | **D** | **N** | **A** | **SA** |
| 4. | I have to deal with numbers. |  |  |  |  |  |
| 5. | Subjects need creative thinking. |  |  |  |  |  |
| 6. | Lack of practicing exercise for these topics. |  |  |  |  |  |
| 7. | I like clinical studies more than epidemiology and biostatistics. |  |  |  |  |  |
| 8. | Lectures were not interesting. |  |  |  |  |  |
| 9. | Lectures were lengthy. |  |  |  |  |  |
| 10. | Lectures were difficult to understand. |  |  |  |  |  |
| 11. | Too many lectures for one day. |  |  |  |  |  |
| 12. | There were no specific references. |  |  |  |  |  |
| 13. | I could not see the relation between statistics and medicine at this level. |  |  |  |  |  |
| 14. | Simply am not interested in the subject. |  |  |  |  |  |
| C | **Attitude towards lecture/lecturers** | **SD** | **D** | **N** | **A** | **SA** |
| 15. | I was treated with respect. |  |  |  |  |  |
| 16. | Work and efforts were acknowledged. |  |  |  |  |  |
| 17. | Lecturer is the source of knowledge. |  |  |  |  |  |
| 18. | Lecturer is the facilitator of instruction & guiding students. |  |  |  |  |  |
| 19. | It is the responsibility of the student to initiate debate/question during lectures. |  |  |  |  |  |

| D | **What type of action you might expect to improve the course** | **SD** | **D** | **N** | **A** | **SA** |
| --- | --- | --- | --- | --- | --- | --- |
| 20. | Give more time for the whole course. |  |  |  |  |  |
| 21. | Introduce this course earlier in year two. |  |  |  |  |  |
| 22. | The lecture should be followed by small group session. |  |  |  |  |  |
| 23. | Carry out shorts exam (quiz) before the progress test to evaluate the understanding of the student. |  |  |  |  |  |
| 24. | Need more practical, workshop to do planning and data collection to have real experience in dealing with data. |  |  |  |  |  |
| 25. | Attendance to be strictly taken during the computer lab session. |  |  |  |  |  |
| 26. | Disconnect the internet during the lab session to avoid distraction. |  |  |  |  |  |
| 27. | Make the module pure for biostatistics and epidemiology, so the attention will not be withdrawn to other subjects. |  |  |  |  |  |
| 28. | Emphasize on using biostatistics in elective courses. |  |  |  |  |  |
| 29. | Provide specific text books for biostatistics and epidemiology. |  |  |  |  |  |
| 30. | I have to study at home before class meetings. |  |  |  |  |  |
| **E** | **At the end of the module** | **SD** | **D** | **N** | **A** | **SA** |
| 31. | I understood the main concepts of Epidemiology & Statistics. |  |  |  |  |  |
| 32. | I realized the relevance of epidemiology & biostatistics to the real health issues. |  |  |  |  |  |
| 33. | I gained confidence in my ability to do basic statistical & epidemiological analysis. |  |  |  |  |  |
| 34. | My skills improved in solving problems. |  |  |  |  |  |
| 35. | I gained skills to read scientific papers. |  |  |  |  |  |
| 36. | I gained skills to design research |  |  |  |  |  |

Note; Questions on section A& E form domain A, other questions stands as they are to form their respective domains.

**Thank you for participation**
